# Low vitamin D status is associated with inflammation in patients with prostate cancer

**DOI:** 10.18632/oncotarget.16195

**Published:** 2017-03-15

**Authors:** Dong-Dong Xie, Yuan-Hua Chen, Shen Xu, Cheng Zhang, Da-Ming Wang, Hua Wang, Lei Chen, Zhi-Hui Zhang, Mi-Zhen Xia, De-Xiang Xu, De-Xin Yu

**Affiliations:** ^1^ Department of Urology, Second Affiliated Hospital, Anhui Medical University, Hefei 230022, China; ^2^ Department of Toxicology, Anhui Medical University, Hefei 230032, China; ^3^ Department of Histology and Embryology, Anhui Medical University, Hefei 230032, China; ^4^ Life Science College, Anhui Medical University, Hefei 230032, China

**Keywords:** prostate cancer, vitamin D deficiency, inflammation, vitamin D receptor, nuclear factor kappa B p65

## Abstract

Vitamin D deficiency has been associated with increased risks of prostate cancer. Nevertheless, the mechanisms remain unclear. The aim of this study was to analyze the association among prostate cancer, vitamin D status and inflammation. Sixty patients with newly diagnosed prostate cancer and 120 age-matched controls were recruited for this study. Vitamin D status was evaluated and serum inflammatory molecules were measured. Serum 25-(OH)D was lower in patients with prostate cancer. Moreover, serum 25(OH)D was lower in patients with severe prostate cancer than patients with mild and moderate prostate cancer. By contrast, serum C-reactive protein (CRP) and interleukin (IL)-8, two inflammatory molecules, were elevated in patients with prostate cancer. Serum 25-(OH)D was negatively correlated with serum CRP and IL-8 in patients with prostate cancer. Additional analysis showed that the percentage of vitamin D receptor positive nucleus in the prostate was reduced in patients with prostate cancer. By contrast, the percentage of nuclear factor kappa B p65-positive nucleus was elevated in patients with prostate cancer. Our results provide evidence that there is an association among prostate cancer, vitamin D deficiency and inflammatory signaling. Inflammation may be an important mediator for prostate cancer progression in patients with low vitamin D status.

## INTRODUCTION

In Western countries, prostate cancer is the most common malignant tumor in men and a major cause of cancer deaths [1]. The incidence of prostate cancer differs between countries due to coverage of prostate-specific antigen (PSA) screening [2]. In China, the incidence of prostate cancer is rapidly increasing and especially in patients with obesity or diabetes [3, 4]. As androgen receptor (AR) signaling is a key pathway for the pathogenesis of prostate cancer, androgen-deprivation therapy remains the principal method for patients with locally advanced and metastatic prostate cancer [5]. Unfortunately, the majority of patients with advanced-stage or metastatic cancer will ultimately progress to castration-resistant prostate cancer [6]. The mechanisms by which prostate cancer progresses to castration-resistant prostate cancer have been studied extensively [7]. Increasing evidence demonstrates that inflammation plays important roles in the pathogenesis of progression to castration-resistant prostate cancer [8].

Vitamin D is a secosteroid hormone and well-known for its classical actions in the maintenance of calcium uptake and bone metabolism [9, 10]. Recently, numerous *in vitro* experiments demonstrated that 1,25-(OH)2D3, the active form of vitamin D, inhibited the growth and differentiation of human prostate cancer cells [11–13]. A double-blinded clinical study found that vitamin D supplementation reduced prostate specific antigen (PSA) level and enhanced survival rate in patients with prostate cancer [14]. On the other hand, vitamin D receptor (VDR) polymorphisms were associated with the incidence of prostate cancer [15, 16]. Several epidemiological reports showed that men with vitamin D deficiency had a higher risk of prostate cancer compared to men with vitamin D sufficiency [17–19]. Nevertheless, the mechanisms through which vitamin D deficiency elevates the risk of prostate cancer remain unclear.

Accumulating evidence demonstrates that vitamin D has an anti-inflammatory activity [20, 21]. Several studies suggest that vitamin D exerts its anti-inflammatory activity through suppressing nuclear factor kappa B (NF-κB) signaling [22–25]. Thus, we hypothesize that vitamin D3 inhibits the incidence and progression of prostate cancer through its anti-inflammatory effect. The present study aimed to investigate whether there was an association among prostate cancer, vitamin D status and inflammation in a hospital-based case-control study. If so, we were to further analyze the expression of VDR and NF-κB p65 subunit in prostatic nuclei.

## RESULTS

### Biochemical characteristics

Biochemical characteristics were analyzed. As shown in Table 1, no significant difference in SBP and DBP, TES, ALT, Cr, UA, TG, TCH, fasting blood glucose, and serum phosphorus was observed between cases and controls. As expected, serum T-PSA was significantly increased in patients with prostate cancer as compared with control subjects (Table 1). In addition, serum LDH was slightly increased in patients with prostate cancer as compared with control subjects (Table 1). Interestingly, serum calcium level was lower in patients with prostate cancer than in controls (Table 1).

**Table 1:** The demographic and biochemical characteristics between cases and controls

	Controls	Cases	*P*
Subjects (n)	120	60	
Years (years)	65.35±7.76	66.35±6.87	0.3987
Height (cm)	166.51±6.75	167.51±5.35	0.3184
Weight (kg)	62.36±5.63	65.82±8.57	0.0014
BMI (kg/m^2^)	21.56±2.57	24.98±2.39	< 0.0001
SBP (mmHg)	129.35±15.86	131.76±13.41	0.3139
DBP (mmHg)	85.45±10.52	87.15±11.26	0.3195
T-PSA (ng/ml)	3.70±2.36	35.70±25.67	< 0.0001
TES (nmol/ml)	10.8±4.81	10.66±5.39	0.8651
ALT (U/L)	13.21±5.89	14.93±6.82	0.0977
AST (U/L)	23.18±3.02	25.09±6.22	0.0270
LDH (U/L)	155.17±22.06	198.65±66.23	< 0.0001
Cr (μmol/L)	84.68±16.52	88.17±16.21	0.1780
UA (mmol/L)	385.35±93.95	391.11±96.33	0.7035
TG (mmol/L)	1.26±1.21	1.22±0.87	0.7999
TCH (mmol/L)	4.22±0.75	4.13±0.67	0.4157
GLU (mmol/L)	5.59±1.21	5.77±1.41	0.3993
Calcium (mmol/L)	2.09±0.13	1.82±0.35	< 0.0001
Phosphorus (mmol/L)	1.12±0.26	1.16±0.19	0.2428

Values are given as the mean±standard deviation.

BMI, body mass index; SBP, systolic blood pressure; DBP, diastolic blood pressure; T-PSA, total prostate specific antigen; Cr, creatinine; UA, uric acid; ALT, alanine aminotransferase; AST, aspartate aminotransferase; LDH, lactate dehydrogenase; TG, triglyceride.

### Association between prostate cancer and vitamin D status

**Figure 1:**
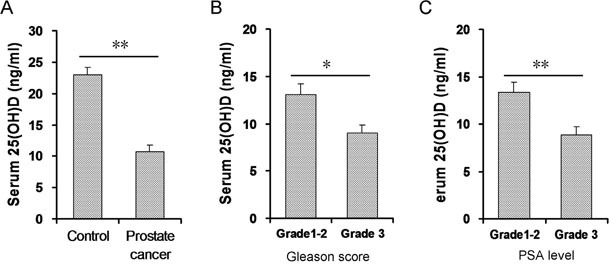
Association between prostate cancer and vitamin D status Serum 25-(OH)D was measured by RIA. **(A)** Serum 25(OH)D level was compared between patients with prostate cancer and controls. (N=60 for patients with prostate cancer; N=120 for controls). **(B)** Patients with prostate cancer were divided into two groups according to Gleason score: mild and moderate prostate cancer (n=34) and severe prostate cancer (n=26). Serum 25(OH)D level was compared between two groups. **(C)** Patients with prostate cancer were divided into two groups according to serum PSA level: mild and moderate prostate cancer (n=31) and severe prostate cancer (n=29). Serum 25(OH)D level was compared between two groups. All data were expressed as means ± S.E.M. *P<0.05; **P<0.01.

Serum 25(OH)D concentration was analyzed in all subjects. As shown in Figure 1A, serum 25(OH)D in patients with prostate cancer was significantly lower than in controls. Multivariable logistic regression analysis was conducted to further evaluate the association between prostate cancer and serum 25(OH)D. As shown in Table 2, there was an inverse association between prostate cancer and serum 25(OH)D (adjusted OR: 0.785; 95%CI: 0.718, 0.858).

**Table 2:** Multivariable logistic regression analysis correlation between prostate cancer and 25(OH)D

Variable	β	Wald	*P*	*OR* (95% *C.I*.)
Unadjusted	-0.235	28.745	<0.001	0.790 (0.725, 0.861)
Adjusted*	-0.242	28.440	<0.001	0.785 (0.718, 0.858)

Note: β: regression coefficient; Wald: Wald chi-square value; *OR*: odds ratio.

*Adjusted for BMI, AST and LDH

### Association between the severity of prostate cancer and vitamin D status

According to Gleason score, prostate cancer patients were categorized into two groups: mild and moderate prostate cancer (Grade 1-2); severe prostate cancer (Grade 3). As shown in Figure 1B, serum 25(OH)D was slightly lower in patients with Grade 3 prostate cancer than patients with Grade 1-2 prostate cancer. Similarly, according to serum PSA level, prostate cancer patients were stratified into two groups: mild and moderate prostate cancer (Grade 1-2); severe prostate cancer (Grade 3). As shown in Figure 1C, serum 25(OH)D was markedly lower in patients with Grade 3 prostate cancer than patients with Grade 1-2 prostate cancer.

### Association between prostate cancer and inflammation

Serum TNF-α, CRP and IL-8 levelswere analyzed in patients with prostate cancer and controls. As shown in Table 3, no significant difference in serum TNF-α level was observed between patients with prostate cancer and controls. Interestingly, serum CRP and IL-8 levels were significantly higher in patients with prostate cancer than in controls (Table 3).

**Table 3:** Association between the severity of prostate cancer and inflammation

	n	TNF-α (pg/ml)	CRP (mg/l)	IL-8 (pg/ml)
All subjects				
Control	120	15.87±1.66	3.01±0.24	68.94±4.46
Prostate cancer	60	15.48±1.66	4.46±0.39**	157.99±21.47**
Grade according to Gleason score				
Grade 1-2	34	14.49±1.36	3.62±0.43	143.56±16.13
Grade 3	26	16.61±2.05	5.13±0.25 †	174.52±24.51
Grade according to PSA levels				
Grade 1-2	32	15.26±1.21	4.22±0.28	134.06±12.25
Grade 3	28	15.68±2.06	4.76±0.37	188.58±19.56 †

All data were expressed as means ± S.E.M. **P<0.01 compare with control; †*P*<0.05 compare with grade 1-2.

### Association between the severity of prostate cancer and inflammation

According to Gleason score, prostate cancer patients were categorized into two groups: mild and moderate prostate cancer (Grade 1-2); severe prostate cancer (Grade 3). Results showed that no statistically significant difference in serum TNF-α and IL-8 levels was observed between two groups (Table 3). Interestingly, serum CRP level was significantly increased in patients with Grade 3 prostate cancer than in patients with Grade 1-2 prostate cancer (Table 3). Similarly, according to serum PSA level, prostate cancer patients were stratified into two groups: mild and moderate prostate cancer (Grade 1-2); severe prostate cancer (Grade 3). Results showed that no significant difference in serum TNF-α and CRP levels was observed between two groups (Table 3). Interestingly, serum IL-8 level was significantly increased in patients with Grade 3 prostate cancer than patients with Grade 1-2 prostate cancer (Table 3).

### Association between vitamin D status and serum inflammatory molecules

**Figure 2:**
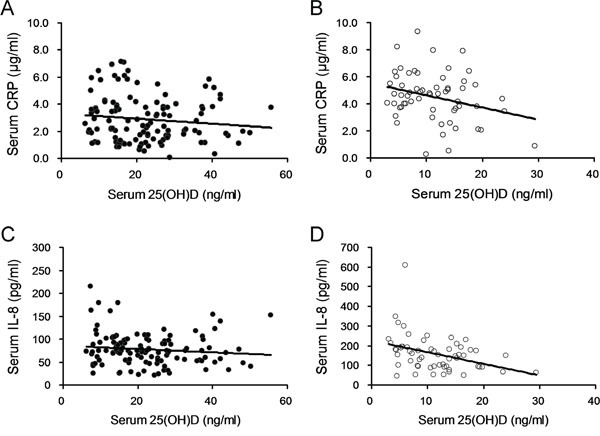
Correlation analysis between serum 25(OH)D and inflammation in patients with prostate cancer and controls **(A)** Correlation analysis between serum 25(OH)D and CRP in control subjects. **(B)** Correlation analysis between serum 25(OH)D and CRP in patients with prostate cancer. **(C)** Correlation analysis between serum 25(OH)D and IL-8 in control subjects. **(D)** Correlation analysis between serum 25(OH)D and IL-8 in patients with prostate cancer.

The correlation between serum 25-(OH)D and CRP levels was analyzed. As shown in Figure 2A, no significant correlation was observed between serum 25-(OH)D and CRP levels in control subjects. Interestingly, serum 25-(OH)D was negatively correlated with serum CRP level in patients with prostate cancer (Figure 2B; *r*=-0.286, *P*<0.05). The correlation between serum 25(OH)D and IL-8 levels was then analyzed. As shown in Figure 2C, no significant correlation was observed between serum 25(OH)D and IL-8 levels in controls. Interestingly, there was an inverse correlation between serum 25(OH)D and IL-8 levels in prostate cancer patients (Figure 2D; *r*=-0.376, *P*<0.01).

### Prostatic NF-κB p65 and VDR in patients with prostate cancer and controls

**Figure 3:**
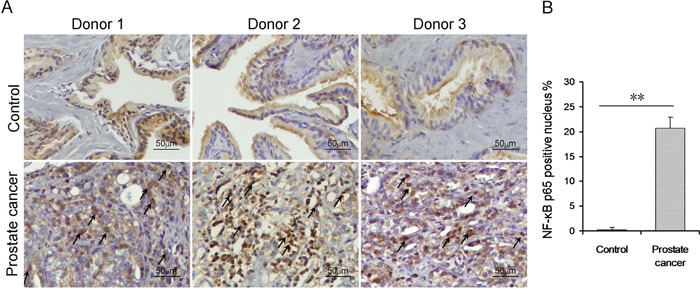
Prostatic NF-κB p65 in patients with prostate cancer and controls **(A)** Prostatic NF-κB p65 was determined using IHC. Arrow indicates NF-κB p65-positive nucleus. **(B)** Rate of prostatic NF-κB p65-positive nuclei was analyzed. All data were expressed as means ± S.E.M. (N=20 for patients with prostate cancer; N=20 for controls). **P<0.01.

**Figure 4:**
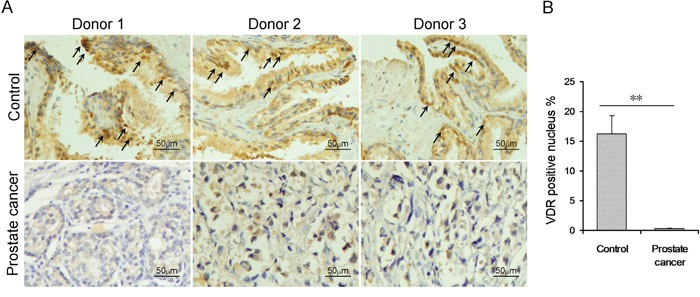
Prostatic VDR in patients with prostate cancer and controls **(A)** Prostatic VDR was determined using IHC. Arrow indicates VDR-positive nucleus. **(B)** Rate of VDR-positive nuclei was analyzed. All data were expressed as means ± S.E.M. (N=20 for patients with prostate cancer; N=20 for controls). **P<0.01.

Prostatic NF-κB p65 was measured in patients with prostate cancer and controls. As shown in Figure 3A, the number of p65-positive nucleus in prostate adenoepithelium was quite small in control subjects. Interestingly, the number of p65-positive nucleus in prostate adenoepithelium was markedly increased in patients with prostate cancer (Figure 3B). Prostatic VDR was then analyzed in patients with prostate cancer and controls. As shown in Figure 4A, numerous VDR-positive nuclei were observed in prostate adenoepithelium of controls. Interestingly, the number of VDR-positive nucleus in prostate adenoepithelium was significantly decreased in patients with prostate cancer (Figure 4B).

## DISCUSSION

The present study analyzed the association among prostate cancer, vitamin D status and inflammation. Our results showed that serum 25-(OH)D was reduced in patients with prostate cancer. By contrast, serum CRP, a marker of systemic inflammation, was elevated in patients with prostate cancer. In addition, serum IL-8 level was also elevated in patients with prostate cancer. These results provide evidence for the first time that low vitamin D status is associated with inflammation in patients with prostate cancer.

Chronic inflammation promotes metastases and progression to castration-resistant prostate cancer [26, 27]. CRP could predict tumor aggressiveness and potential treatment efficacy in patients with prostate cancer [28]. According to an early report, CRP is an independent prognostic factor for overall survival of patients with castration-resistant prostate cancer treated with docetaxel [29]. A recent study showed that elevated CRP level was associated with poor prognosis in prostate cancer patients treated with radiotherapy [30]. Accumulating data demonstrate that IL-8 is expressed by prostate cancer cells in which it contributes to increased angiogenesis, metastasis, and progression toward castration and chemotherapy resistance [31, 32]. An early study showed that IL-8 promoted androgen-independent proliferation of prostate cancer cells via inducing androgen receptor expression and activation [34]. Additional report indicated that IL-8 attenuated TRAIL- and chemotherapy-induced apoptosis through transcriptional regulation of c-FLIP in prostate cancer cells [34]. A recent study found that IL-8 sustained cell survival in PTEN-deficient prostate carcinoma [35]. The present study analyzed the levels of serum 25-(OH)D and inflammatory molecules in patients with different grades of prostate cancer. Our results showed that serum 25-(OH)D level was lower in patients with severe prostate cancer than in patients with mild and moderate prostate cancer. By contrast, serum CRP and IL-8 levels were higher in patients with Grade 3 prostate cancer than in patients with Grade 1-2 prostate cancer. These results suggest that low vitamin D status is associated with inflammation and the progression of prostate cancer.

Increasing evidence indicates that vitamin D has an anti-inflammatory activity [20]. According to an early report, vitamin D inhibited IL-8 production in TNF-α-stimulated human dermal fibroblasts [36]. A recent study found that vitamin D suppressed IL-8 production in bacterial virulence factors-stimulated monocyte-derived macrophages [37]. The present study analyzed the association between vitamin D status and serum inflammatory molecules in patients with prostate cancer. Although no significant correlation was observed between serum 25-(OH)D and inflammatory molecules in control subjects, serum 25-(OH)D was negatively correlated with serum CRP and IL-8 levels in patients with prostate cancer. These results suggest that inflammation may be a key mediator for prostate cancer progression in patients with low vitamin D status.

Several studies demonstrate that vitamin D blocks NF-κB activation [24, 38, 39]. Indeed, IL-8, responsible for the androgen-independent growth of advanced prostate cancer, is a downstream target of NF-κB signaling [40]. On the other hand, NF-κB activation promotes tumor cell survival through its anti-apoptotic mechanism in prostate cancer [41]. In addition, NF-κB activation correlates with metastasis and prostate cancer progression to castration-resistant prostate cancer [42, 43]. The present study measured prostatic VDR and NF-κB p65 subunit in patients with prostate cancer. As expected, nuclear VDR in the prostate was reduced in patients with prostate cancer. By contrast, prostatic NF-κB signaling was activated in patients with prostate cancer. These results provide a mechanistic explanation for the association between prostate cancer and vitamin D deficiency.

The present study has several limitations. First, the present study did not observe whether vitamin D deficiency and inflammation promotes metastasis and progression of prostate cancer. Second, the present study did not clarify the mechanism through which vitamin D deficiency regulates prostatic NF-κB signaling and inflammation in patients with prostate cancer. Thus, additional study is required to investigate whether prostatic inflammation promotes progression to castration-resistant prostate cancer in patients with low vitamin D status. In addition, the mechanism through which vitamin D deficiency regulates prostatic inflammation and progression to castration-resistant prostate cancer needs to be explored in an *in vitro* experiment.

In summary, the present study investigated the association among prostate cancer, vitamin D status and inflammation. Our results showed that serum 25-(OH)D was decreased in patients with prostate cancer. By contrast, serum CRP and IL-8 were increased in patients with prostate cancer. We found that prostatic VDR signaling was attenuated in patients with prostate cancer. By contrast, prostatic NF-κB signaling was activated in patients with prostate cancer. Our results provide evidence that there is an association among prostate cancer, vitamin D deficiency and inflammatory signaling.

## MATERIALS AND METHODS

### Study participants

This was a hospital-based case-control study using data from the Second Affiliated Hospital of Anhui Medical University in China between January 2013 and December 2014. In the present study, total 60 newly diagnosed patients with prostate cancer were recruited as cases. Prostate cancer were confirmed by histopathology. Controls were recruited from men undergoing physical examination at the Second Affiliated Hospital of Anhui Medical University. Each case was matched with two controls with regard to age (within 2 years). Potential controls with malignant tumor were excluded from this study. Serum samples of all cases and controls were collected at same season and stored at -80°C. The demographic characteristics between cases and controls were shown in Table 1. For measurement of prostatic VDR and NF-κB p65, controls were recruited from histologically confirmed patients with benign prostatic hyperplasia at the Second Affiliated Hospital of Anhui Medical University. Each case was matched with one control with regard to age. All samples from cases and controls were collected at same season. In this study, total 20 controls and 20 cases were recruited for measurement of prostatic VDR and NF-κB p65. The present study obtained ethics approval from the ethics committee of Anhui Medical University. Oral and written consents were obtained from all subjects.

### Grade of prostate cancer

In the present study, Gleason score and PSA level were used to evaluate the severity of prostate cancer, respectively. According to Gleason score, prostate cancer patients were categorized into two groups: mild and moderate prostate cancer (Grade 1-2; Gleason score ≤ 7); severe prostate cancer (Grade 3; Gleason score: 8-10). Similarly, according to serum PSA level, prostate cancer patients were stratified into two groups: mild and moderate prostate cancer (Grade 1-2; PSA ≤20 ng/ml); severe prostate cancer (Grade 3; PSA > 20 ng/ml) [44, 45].

### Clinical and biochemical measurements

Demographic and clinical characteristics were collected by a standard questionnaire, including age, education, occupation, history of cigarette smoking, alcohol intake and medications. Height, weight, systolic blood pressure (SBP) and diastolic blood pressure (DBP) were from record of physical examinations. Body mass index (BMI) was calculated. Venous blood was drawn in the morning with more than 8 hours fasting. Serum total prostate specific antigen (T-PSA), creatinine (Cr), uric acid (UA), alanine aminotransferase (ALT), aspartate aminotransferase (AST), lactate dehydrogenase (LDH), triglyceride (TG), serum calcium, serum phosphorus, and fasting blood glucose were measured with automatic biochemical analyzer (KHBZY-1200).

### 25-(OH)D measurement

Serum 25-(OH)D concentration, as the most stable circulating form of this molecule, was measured by radioimmunoassay using a kit from Diasorin (DiaSorin Inc, Stillwater, MN, USA) following manufacturer's instructions [46]. Serum 25-(OH)D concentration is expressed as ng/ml. Lower than 15 ng/mL of 25-(OH)D was defined as vitamin D deficiency [47].

### Enzyme-linked immunosorbent assay (ELISA)

Commercial ELISA kits (R&D Systems, Abingdon, Oxon, UK) were used to measure serum TNF-α, C-reactive protein (CRP) and IL-8 according to the manufacturer's protocol.

### Hematoxylin-eosin (HE) and immunohistochemistry

Human prostate tissues from prostate cancer cases and benign prostatic hyperplasia controls were fixed in 4% formalin and embedded in paraffin according to the standard procedure. Paraffin embedded tissues were cut 5 μm thick and stained with HE for histopathological analysis. For immunohistochemistry, paraffin-embedded prostate sections were deparaffinized and rehydrated in a graded ethanol series. After antigen retrieval and quenching of endogenous peroxidase, sections were incubated with NF-κB p65 or VDR monoclonal antibodies (Santa Cruz, CA, USA, 1:200 dilution) at 4°C overnight. The color reaction was developed with HRP-linked polymer detection system and counterstaining with hematoxylin. The number of positive nuclei and total nuclei were counted in twelve randomly selected fields from each slide at a magnification of ×400. The percentage of positive nuclei was calculated as a percentage of positive nuclei relative to the total nuclei.

### Statistical analysis

SPSS version 16.0 statistical package was used for statistical analyses. The difference in continuous variables between two independent groups was compared using two independent sampling t test or the Mann-Whitney U-test. Comparative analyses of categorical variables were carried out by the chi-square test. Multivariable logistic regression was carried out to identify the associations between 25(OH)D and prostate cancer. The relationship between 25(OH)D concentration and IL-8 and CRP levels were analyzed using scatter plots and linear correlation. A *P* value of less than 0.05 was considered statistically significant.
